# Velusetrag rescues GI dysfunction, gut inflammation and dysbiosis in a mouse model of Parkinson’s disease

**DOI:** 10.1038/s41531-023-00582-1

**Published:** 2023-10-02

**Authors:** Jessica Grigoletto, Fabiana Miraglia, Laura Benvenuti, Carolina Pellegrini, Sara Soldi, Serena Galletti, Antonino Cattaneo, Emilio Merlo Pich, Maria Grimaldi, Emanuela Colla, Loredana Vesci

**Affiliations:** 1 Bio@SNS Laboratory, Scuola Normale Superiore, Piazza dei Cavalieri 7, 56126 Pisa, Italy; 2 Department of Clinical and Experimental Medicine, University of Pisa, Via Roma 55, 56126 Pisa, Italy; 3 Unit of Histology and Medical Embryology, Department of Clinical and Experimental Medicine, University of Pisa, Via Roma 55, 56126 Pisa, Italy; 4 AAT Advanced Analytical Technologies Srl, via P. Majavacca 12 – 29017, Fiorenzuola d’Arda (PC), Italy; 5 Neurotrophins and Neurodegenerative Diseases Laboratory, Rita Levi-Montalcini European Brain Research Institute, Viale Regina Elena 295, Rome, 00161 Italy; 6 Corporate R&D, Alfasigma S.p.A., Via Pontina km 30.400, 00071 Pomezia (Rome), Italy; 7 Department of Human Sciences and Promotion of Quality of Life, San Raffaele Open University, Via Val Cannuta 247, 00166 Rome, Italy

**Keywords:** Parkinson's disease, Cellular neuroscience

## Abstract

In patients with Parkinson’s disease (PD), constipation is common, and it appears in a prodromal stage before the hallmark motor symptoms. The present study aimed to investigate whether Velusetrag, a selective 5‑HT4 receptor agonist, may be a suitable candidate to improve intestinal motility in a mouse model of PD. Five months old PrP human A53T alpha-synuclein transgenic (Tg) mice, which display severe constipation along with decreased colonic cholinergic transmission already at 3 months, were treated daily with the drug for 4 weeks. Velusetrag treatment reduced constipation by significantly stimulating both the longitudinal and circular-driven contractions and improved inflammation by reducing the level of serum and colonic IL1β and TNF-α and by decreasing the number of GFAP-positive glia cells in the colon of treated mice. No significant downregulation of the 5-HT4 receptor was observed but instead Velusetrag seemed to improve axonal degeneration in Tgs as shown by an increase in NF-H and VAChT staining. Ultimately, Velusetrag restored a well-balanced intestinal microbial composition comparable to non-Tg mice. Based on these promising data, we are confident that Velusetrag is potentially eligible for clinical studies to treat constipation in PD patients.

## Introduction

Parkinson’s Disease (PD) is a multi-organ proteinopathy associated with the accumulation of alpha-synuclein (αS) deposits through the CNS and PNS^1^. PD is clinically characterized by an advanced stage when deposition of αS in Lewy bodies (LBs) and degeneration of dopaminergic neurons in the nigrostriatal pathway result in the occurrence of hallmark motor symptoms^2^. Yet, decades earlier an asymptomatic prodromal phase with a wide range of non-motor symptoms, such as hyposmia, sleep disorders, depression, and constipation, begins to manifest^3^. The main cause of constipation in PD in most cases is a slower colonic transit^4–6^ and this involves LB pathology. Peripheral αS aggregates have been observed along the whole gastrointestinal (GI) tract in patients, in both the submucosal and myenteric plexi of the colon as well as the salivary glands, lower parts of the esophagus and the stomach^7–10^. Loss of dopaminergic neurons in the enteric nervous system (ENS) of PD patients as well as the role of dopamine (DA) in the gut are still under debate, as they represent only <3% of the total enteric neuron population^11–13^, whereas degeneration of intestinal cholinergic innervation and impairment of cholinergic transmission, seem to be primarily responsible for delaying colonic transit time in PD patients^14,15^. Notably, DA inhibits acetylcholine (ACh) release and consequently intestinal motility, by binding to its D2 presynaptic receptor in myenteric neurons^16^. This explains why constipation in PD is further worsened by antiparkinsonian medications, first and foremost by levodopa itself.

Although serotonin (5-HT) is best known as a neurotransmitter fundamental for the CNS, 95% of the body’s serotonin is produced in the intestine by enterochromaffin cells (EC) of the GI mucosa and enteric neurons^17^. The enteric 5-HT released by EC cells, activates 5-HT receptors on intrinsic and extrinsic afferent fibers of the lamina propria, triggering a plethora of reflex responses^18^ that are crucial for GI motility, inflammation, and ENS neurogenesis. For instance, stimulation of 5-HT4 receptor, leads to an increase in the peristaltic reflex pathways by acting presynaptically on nerve terminals within the myenteric and submucosal plexi to enhance the release of ACh from myenteric neurons, therefore stimulating longitudinal and circular muscle contractility, and counteracting constipation^19–21^. In addition, since 5-HT4 receptors are also expressed on colonic epithelial cells, their stimulation promotes mucus discharge from goblet cells and chloride secretion by enterocytes that in turn can alleviate constipation and GI dysfunction^21^. Therefore, over the years, several molecules agonist of the 5-HT4 receptor have been developed and tested for the treatment of chronic constipation.

In this study, we used Velusetrag, also known as TD-5108, a highly selective 5-HT4 receptor agonist. This compound has demonstrated robust efficacy and good tolerance in both healthy volunteers and patients with chronic idiopathic constipation^22,23^. Administration of a single daily dose for 4 weeks produced a dose-related prokinetic activity, including an increase in gastric emptying, intestinal and colonic transit, and stool production^22^. Velusetrag has good potency (pEC50 = 8.3), and a high intrinsic activity on human and rodent receptors in GI tissue and it is generally safe^24^. Interestingly, this compound has been shown to induce cognitive improvement in animal models of Alzheimer’s Disease^25^ and PD^26^, therefore it is also under consideration as a potential candidate to cure dementia (NCT01467726 on ClinicalTrials.gov). Because of this double effect of Velusetrag on the ENS and CNS, it is imperative to investigate its actions in models where both types of symptomatology and pathology are temporally well distinct.

Therefore, we used the PrP human A53T αS transgenic (Tg) mice, a PD mouse model where motor and non-motor symptoms are spatially and temporally separated, to assess the prokinetic effect of Velusetrag in the context of PD. We used 5-month-old Tg mice. At this age, these mice already show a 50% reduction in colonic peristalsis with a concomitant 50–60% decrease in cholinergic transmission resulting in 2 hours delay in food transit along the GI tract^27^. We found that 4 weeks of Velusetrag treatment greatly and positively affected GI dysfunction, colonic inflammation and re-established a well-balanced microbiota. In addition, Velusetrag seemed to have a positive effect on axonal degeneration in the distal colon of treated A53T mice, as shown by an increase in NF-H and VAChT staining. These results indicate that Velusetrag is potentially eligible for clinical studies to treat chronic constipation in PD patients.

## Results

### Treatment with Velusetrag improves colon motility without altering fecal output

**Fig. 1:**
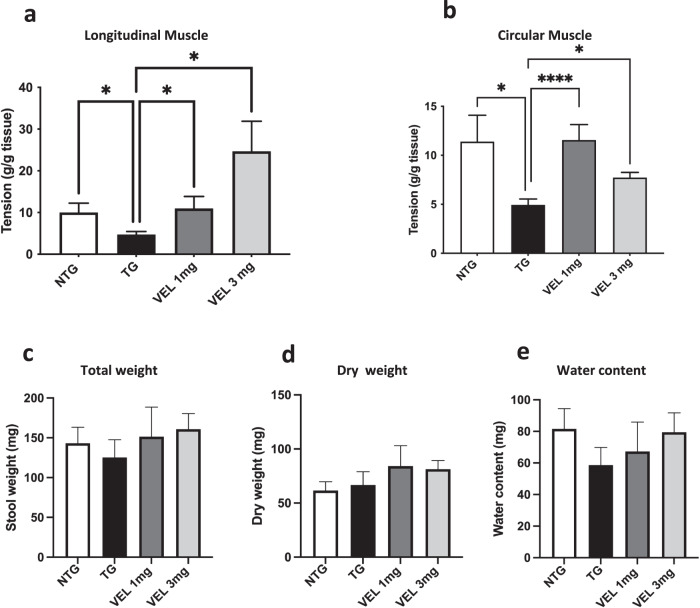
Colon dysmotility is abolished by Velusetrag treatment in the A53T Tg mice. GI dysfunction in the A53T mice is abolished by Velusetrag treatment. **a**, **b** Analysis of myogenic response in Tgs mice and Ntg littermates showed a reduction in gut dysmotility for the longitudinal (**a**) and circular-driven (**b**) type of contractions after Velusetrag treatment compared to Tg animals treated with vehicle. Distal colon stripes were mounted on an isometric force transducer, stimulated with a pair of coaxial platinum electrodes, and mechanical activity was recorded as a measure of tension. Graphs show mean value of tension for each group of mice. Values are expressed as raw data and are given as the mean ± SEM (*p < 0.05, ****p < 0.0001, *n* = 4–5 per group, Brown-Forsythe and Welch ANOVA tests for the longitudinal muscle and Kruskal–Wallis ANOVA test for circular muscle). **c**–**e** Fecal output is not affected by Velusetrag administration. Pellets were collected a week before the sacrifice and weight before and after dehydration. Water content was calculated as the difference between total and dry weight. Values on the graph are expressed as raw data and are given as the mean ± SEM (*n* = 7–14, One-way ANOVA, Tukey post hoc test).

Since the primary described effect of Velusetrag is on intestinal constipation, analysis of colon motility and fecal output was performed in all groups of mice at 6 months of age after treatment completion as initial read out to assess target engagement. Recording of intestinal contractions on ex-vivo colonic sections induced by external electrical stimulation confirmed a significant decrease in colonic movements of the longitudinal and circular muscles in Tg mice compared to Ntgs as previously described for this mouse line^27^ (Fig. 1a, b). Significantly Tgs treated with Velusetrag showed a consistent increase in colonic motility for the longitudinal muscle in a dose-dependent manner (the average was 10.97±2.8 g/g tissue for 1 mg and 24.67±7.2 for 3 mg vs 4.7±0.7 for Tgs treated with water, *p < 0.05) and for the circular muscle (average 11.55±1.5 g/g tissue for 1 mg and 7.7±0.52 for 3 mg vs 4.9±0.6 for Tgs treated with water, *p < 0.05) compared with the Tg mice that only receive sterile water. Such a drastic rescue effect translated only in a slight increase, although not significant, of pellets in terms of total and dry weight or in the stool water content in groups of animals treated with the 5-HT4 agonist (Fig. 1c–e). Velusetrag treatment was well-tolerated at the chosen doses because no significant change in body weight or motor function and coordination between all groups during treatment and Tg mice was found (Supplementary Fig 1). On the whole, these results demonstrate the prokinetic effect of Velusetrag on colon motility for both the longitudinal and circular-driven type of movements with promotion of intestinal contractility already at low dosage.

### Velusetrag treatment improves axonal degeneration in the colon of Tg mice

**Fig. 2:**
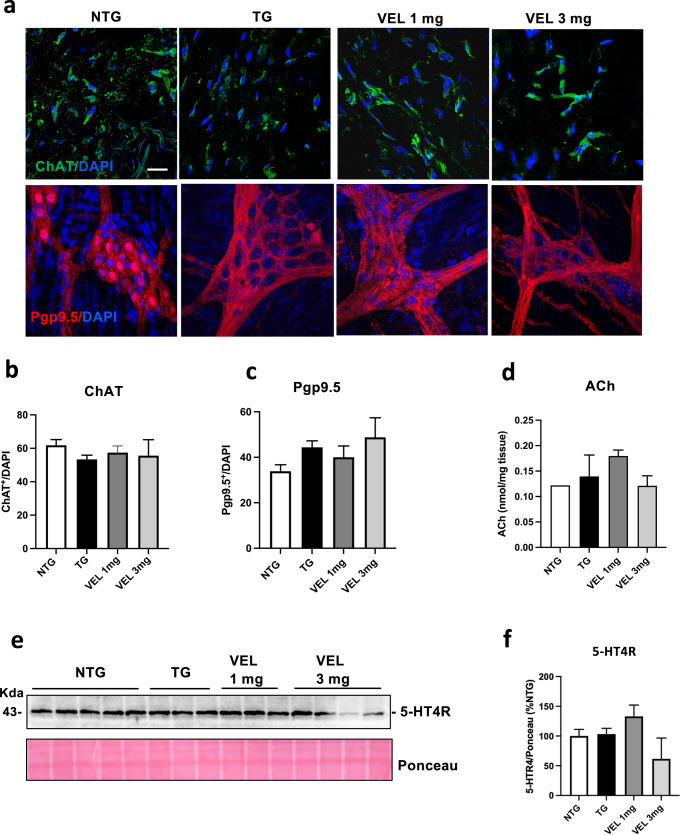
Absence of neuronal loss in the distal colon of Tg mice, including cholinergic neurons after Velusetrag treatment. Evaluation of the ENS network. **a** Whole-mount sections obtained from distal colon of A53T αS Tg treated either with vehicle or 1 or 3 mg Velusetrag and Ntg littermates were immunostained with anti-Pgp9.5 and anti-ChAT antibodies. Nuclei were labeled with DAPI. Images were acquired by using a Zeiss LSM 900 airyscan 2 confocal microscope and a 40x objective. Bar = 10 μm. Neuronal count for Pgp9.5^+^ or ChAT^+^ cells was determined using Image J software. Each signal was normalized with the corresponding number of nuclei. **b**, **c** Graphs of ChAT^+^ (**b**) or Pgp9.5^+^ (**c**) neurons showing that there are no evident neuronal population changes in Tg animals compared to Ntg littermates or after Velusetrag treatment. Data on graphs are expressed as mean ± SEM (*n* = 10, one-way ANOVA, Tukey post hoc test for Pgp9.5^+^ or ChAT^+^ neurons). **d** Colonic ACh was determined through ELISA from lysates of distal colon of Tgs treated with vehicle or Velusetrag and Ntg littermates. Data on the graph are expressed as mean ± SEM (*n* = 4–5, One-way ANOVA, Tukey post hoc test). **e**, **f** Expression level of the 5-HT4 receptor (5-HT4R) remains unaltered during Velusetrag treatment. WB analysis of total fractions of distal colon obtained from Tgs mice treated with Velusetrag or vehicle and Ntg littermates showed no desensitization of 5-HT4R after treatment (**e**). The densitometry of immunoblots was determined with ImageLab software. Data on graph (**f**) shows quantitative analysis of 5-HT4R abundance and are expressed as mean ± SEM (*n* = 4–5 per group, One-way ANOVA, Tukey post hoc test).

**Fig. 3:**
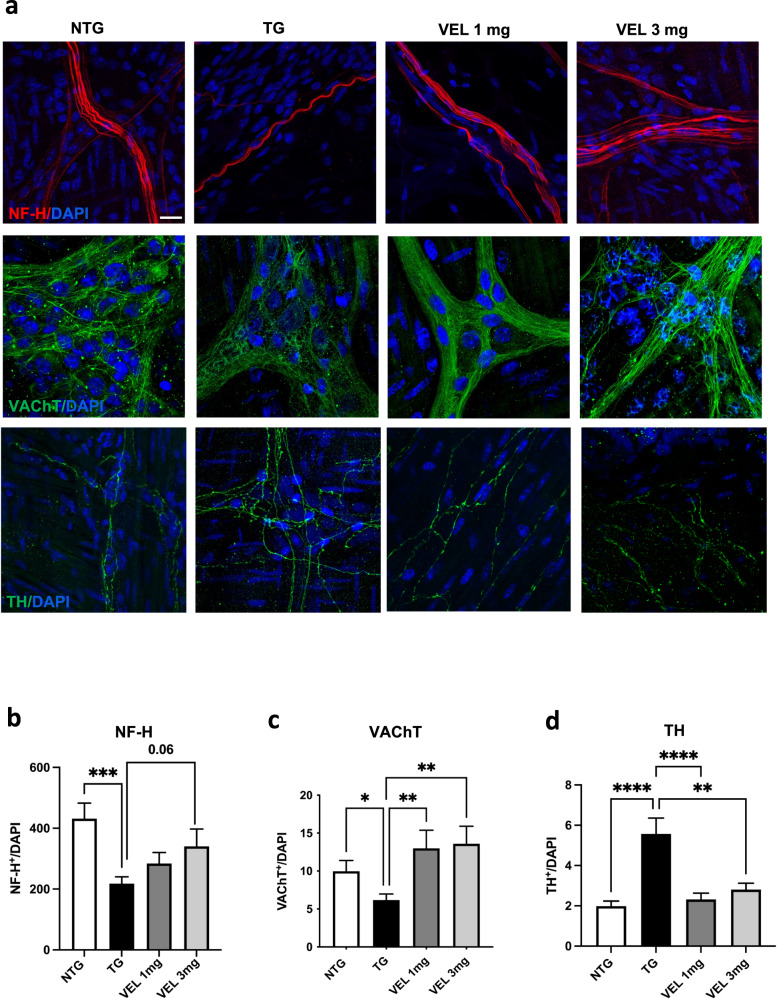
Velusetrag improves axonal degeneration in the colon of Tg mice. Whole mount sections obtained from the distal colon of A53T αS Tg-treated either with vehicle or 1 or 3 mg Velusetrag and Ntg littermates were immunostained with anti-NF-H, anti-VAChT, or anti-TH antibodies to analyze axonal damage. **a** Representative fluorescent images of all groups were acquired by using a Zeiss LSM 900 airyscan 2 confocal microscope and a 40x objective. Nuclei were labeled with DAPI. Bar = 10 μm. Mean fluorescence of NF-H, VAChT, and TH staining was determined using Image J software. Each signal was normalized with the corresponding number of nuclei. **b**, **c** Graphs of NF-H (**b**) or VAChT ^+^ (**c**) neurons showed a significant axonal degeneration in Tg mice treated with vehicle compared to age-matched Ntgs, including cholinergic terminals, whereas mice treated with Velusetrag, showed a dose-dependent positive trend in the case of NF-H (*p* = 0.06), particularly at 3 mg or for both doses in the case of VAChT (**p < 0.01 Tg vs Velusetrag 1 mg and 3 mg). **d** Graph of TH^+^ neurons showed an unexpected increase in TH expression in Tg animals treated with vehicle compared to controls, that was lowered to levels similar to Ntgs by Velusetrag administration (**p < 0.01, ****p < 0.0001). All data on graphs are expressed as mean ± SEM (*n* = 10–15 images per each group, Brown–Forsythe and Welch one-way ANOVA followed by unpaired *t* with Welch’s correction for NF-H, Kruskal–Wallis ANOVA followed by Uncorrected Dunn’s test for VAChT and one-way ANOVA followed by Fisher’s LSD test for TH).

5-HT mechanism of action in the intestine is exerted through the activation of cholinergic innervation that dictates contractile movements^17–19^. The A53T αS mice show a defect in cholinergic transmission in the colon that is responsible for slow, colonic contractions and constipation^27^. Since such abnormalities could suggest the onset of a neurodegenerative process of specific neuronal populations in this area, we investigated anatomical aspects of the ENS in the colon after Velusetrag treatment by assessing the amount of colonic ACh in relation to the presence of possible changes in the cholinergic network and in the whole neuronal population. Immunohistochemistry analysis of whole mount preparations from the distal colon of A53T αS mice and age-matched Ntgs did not show significant differences in the level of ChAT^+^ neurons as well as in the total number of ENS neurons stained with Pgp9.5. No significant differences were also found after Velusetrag administration (Fig. 2a–c). Similarly, when tissue ACh was assayed, no consistent evidence of changes in the total amount of this neurotransmitter were found in Tgs compared to Ntg controls, even after Velusetrag treatment (Fig. 2d). At the same time, no differences in the expression level of Velusetrag pharmacological target, the 5-HT4 receptor, were found in all groups examined (Fig. 2e, f), although the 3 mg group showed a non-significant decrease. Therefore, no evident signs of neuronal loss including cholinergic neurons that could explain the reduced colonic motility or 5-HT4 receptor downregulation was evident in our Tg animal model with or without the administration of the pharmacological treatment. Since recent evidence suggested that 5-HT4 agonists can induce neuronal differentiation and neurites elongation after injury in guinea pigs^28^, we immunostained the distal colon sections with NF-H, a protein belonging to a class of cytoskeletal components of axons, that together with neurofilament light (NF-L) and medium (NF-M) chain are widely investigated as potential biomarkers of axonal insult in neurological disorders^29^ (Fig. 3a, b). Surprisingly Tg animals treated with the vehicle showed a drastic reduction in NF-H content compared to Ntg littermates (mean fluorescence intensity was 218.14±22 for Tgs vs 431.96±51 for Ntgs, ***p < 0.001) a pattern that seemed positively changed, in a dose-dependent manner, by administration of Velusetrag (mean 340.8±56.7, VEL 3 mg, *p* = 0.06). Similarly, analysis of VAChT, a marker of cholinergic presynaptic terminals^30,31^, showed a significant decrease in Tg mice (mean fluorescence 9.972±1.4 for Ntgs vs 6.18±0.79 for Tgs, *p* < 0.05) that was recovered after Velusetrag administration (mean fluorescence 12.99±2.39 for 1 mg and 13.6±2.30 for 3 mg) (Fig. 3a, c). Together these results suggest that intestinal cholinergic deficit in αS Tg animals is possibly ascribable to an axonal degeneration rather than a systemic neuronal loss and that treatment with Velusetrag improves the colonic axonal network. Recently, ex-vivo analysis of mouse distal colon preparations highlighted that DA could contribute to GI motility by acting as an inhibitory signal and reducing ACh release^16^. Therefore, analysis of whole mount distal colon sections for the presence of TH, the rate-limiting enzyme involved in the first step of DA production, was carried out in all groups (Fig. 3a, d). Unexpectedly and surprisingly, Tg mice treated with only vehicle showed a significant increase in TH expression compared to Ntg littermates (mean fluorescence was 5.57±0.78 for Tgs vs 1.982±0.25 for Ntgs). Moreover, Velusetrag treatment at both doses re-established TH expression in Tgs at levels similar to the Ntg group, an unexpected effect for this drug that would need further investigation. Together these results suggest that multiple aspects including possibly DA or other catecholamines modulation could concur in inhibiting colon motility in the PrP Tg mouse model.

### Velusetrag stimulates the activation of AKT pro-differentiation signaling

**Fig. 4:**
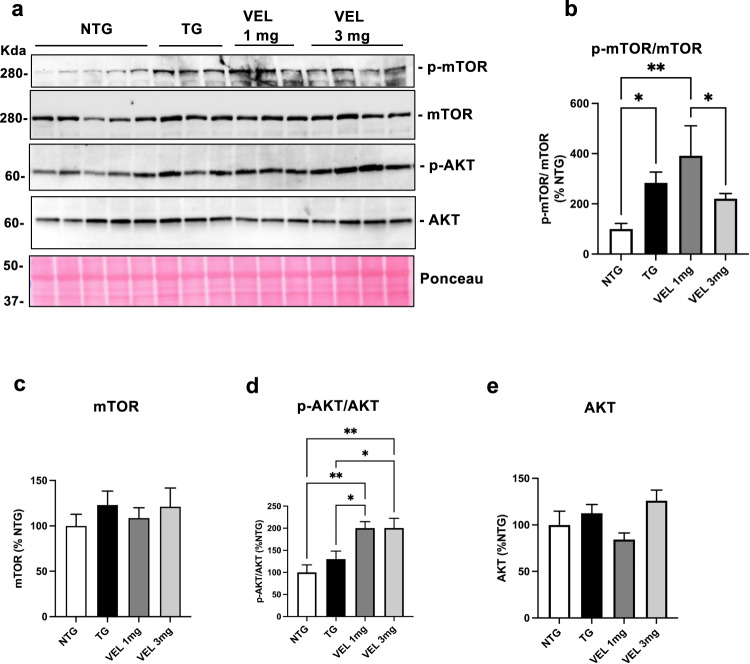
Activation of AKT/mTOR cellular signaling in Tg mice after Velusetrag treatment. Activation of AKT/mTOR signaling was evaluated in the distal colon of Tg mice treated with Velusetrag or vehicle and Ntg littermates. **a** Samples of total fractions of distal colon obtained from A53T αS Tg and control mice were analyzed by WB and immunoblotted with anti-phospho-mTOR, anti-mTOR, anti-phospho-AKT and anti-AKT antibodies. Protein bands intensity was determined using ImageLab and was normalized with the total protein load visualized through Ponceau staining. **b**–**e** Graphs showing densitometric analysis of blots obtained in **a**. Phospho-mTOR and phospho-AKT are expressed as the ratio between the phosphorylated protein (**b**, **d**) and the corresponding total protein (**c**, **e**). AKT was found to be significantly phosphorylated by Velusetrag treatment. On the contrary mTOR was greatly activated in Tg animals treated with vehicle compared to Ntgs but the 5-HT4 agonist regimen did not further increase its phosphorylation. Data on graphs are expressed as % to Ntgs and given as the mean ± SEM (*n* = 4–5, One-way ANOVA, Fisher’s LSD post hoc test, **p < 0.01, *p < 0.05).

To further investigate Velusetrag molecular effect on axonal degeneration in the colon, we analyzed the AKT/mTOR signaling, a cell pathway involved in promoting cell growth, division, proliferation, and differentiation in physiological and pathological conditions^32^. Activation of AKT/mTOR signaling through their phosphorylation was determined in total lysates of distal colon obtained from treated Tg and Ntg animals. AKT phosphorylation was found to be significantly increased after Velusetrag treatment compared to Tgs treated only with water (Fig. 4a, d, e). The maximum response was already reached with the lowest dose of Velusetrag (mean intensity was 200.35±14.56 for 1 mg and 200.5±21.8 3 mg vs 130.11±18.3 for Tgs). On the contrary, a downstream effector of AKT, mTOR, was already significantly phosphorylated in αS Tg animals treated with water compared to Ntg littermates (283.9±42.8 vs 100±22.3), confirming previous data obtained in this and other models of α-synucleinopathy where overexpression of αS was sufficient to activate mTOR^33,34^. Moreover both doses of Velusetrag did not result in an additional activation of phospho-mTOR (Fig. 4a–c). Thus, while activation of phospho-mTOR seems to be related to an increased inhibition of autophagy by overexpression of αS and in particular of the A53T mutant, in cell and mouse models of PD^33,34^, it is possible that other downstream players of AKT may be important in mediating Velusetrag’s effect on axonal degeneration.

### Velusetrag treatment improves peripheral and colonic inflammation in the A53T αS mice

**Fig. 5:**
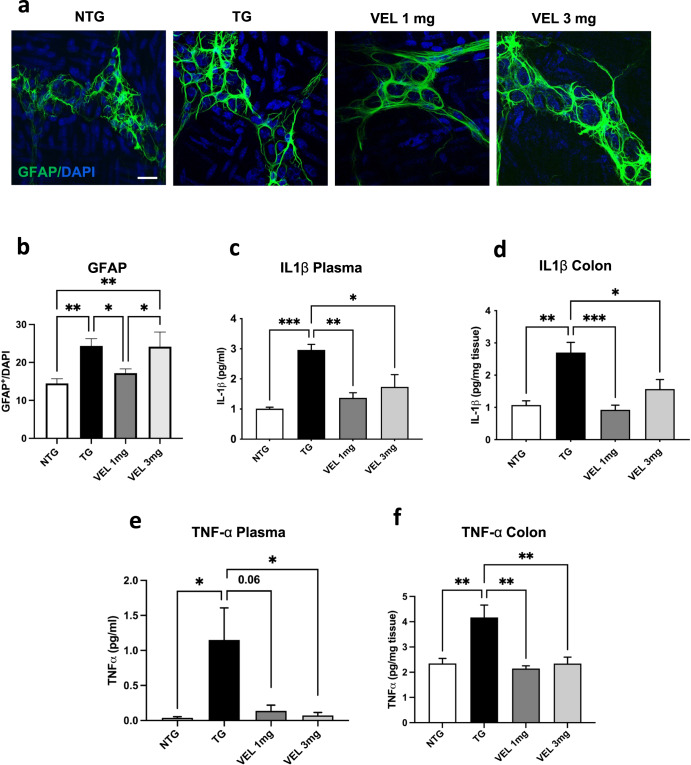
Velusetrag rescues peripheral and colonic inflammation. Administration of Velusetrag abolishes peripheral and gut inflammation by reducing the level of proinflammatory cytokines in plasma and in colon and by decreasing the number of activated glia cells in the colon wall. **a** Whole-mount sections obtained from distal colon of A53T αS Tg treated either with vehicle or 1 or 3 mg Velusetrag and Ntg mice were stained with anti-GFAP antibody. Nuclei were counterstained with DAPI. Images were acquired using a Zeiss LSM 900 airyscan 2 confocal microscope and a 40x objective. Bar = 10 μm. **b** GFAP^+^ glia cells level was counted using Image J and normalized with the corresponding DAPI staining. Immunostaining analysis showed an increase of GFAP^+^ cells in Tgs treated with vehicle compared with Ntg littermates. Administration of 1 mg of Velusetrag normalized significantly GFAP^+^ signal, while 3 mg dose had no clear effect. Data on graphs are expressed as mean ± SEM (*n* = 10, One-way ANOVA, Fisher’s LSD post hoc test, **p < 0.01, *p < 0.05. **c**–**f** Peripheral (**c**, **e**) and colonic (**d**, **f**) level of proinflammatory cytokines after Velusetrag treatment. ELISA assays of IL1β and TNF-α showed a rapid decline of pro-inflammatory cytokines in plasma and in colon after treatment with Velusetrag. Data on graphs are expressed as the mean ± SEM (*n* = 4–5, One-way ANOVA, Tukey post hoc test for IL1β and Brown-Forsythe and Welch ANOVA tests for TNF- α, *p < 0.05; **p < 0.01; ***p < 0.001).

Since the presence of colonic and peripheral inflammation already in the prodromal phase before motor deficit is a salient feature of the αS A53T line used in this study, we assessed the level of colonic and peripheral pro-inflammatory cytokines (IL1β and TNF-α) and the level of enteric glia using GFAP as specific marker. In agreement with our previous work^35^, we found that the A53T mice have increased levels of GFAP^+^ cells in the distal colon compared to Ntg age-matched littermates (mean cell count 24.35±1.9 for Tgs vs 14.5±1.1 for Ntgs, **p < 0.01) (Fig. 5a, b). This pattern agrees with the concomitant increased level of tissue and circulating pro-inflammatory cytokines found in the Tgs treated with vehicle (Fig. 5c–f). Remarkably, the administration of Velusetrag greatly reduced colonic and peripheral inflammation. The number of GFAP^+^ cells was significantly decreased after administration of 1 mg of Velusetrag compared to Tgs that only received the vehicle (mean 17.19±1.1 vs 24.35±1.9, *p < 0.05). Unexpectedly the 3 mg dose had scarce effect. Instead, on the substantial reduction of tissue and circulating pro-inflammatory cytokines, we found a significant potent counteraction induced by the Velusetrag treatment. For example, IL1β level in plasma went from 2.96±0.18 pg/ml in Tgs treated with vehicle to 1.37±0.17 pg/ml after Velusetrag 1 mg and 1.73±0.4 pg/ml after Velusetrag 3 mg, whereas in colonic tissue, IL1β levels were reduced from 2.7±0.3 pg/mg to 0.92±0.14 pg/mg after Velusetrag 1 mg and 1.57±0.29 pg/mg after Velusetrag 3 mg. Similar effect was seen for TNF-α abundance. In addition, for both cytokines, no direct dose-dependent effect was observed since the level of IL1β and TNF-α was already at a minimum low with the lowest dose of Velusetrag (1 mg) and did not further decrease with the 3 mg dose (Fig. 5c–f). Taking together these data suggest that Velusetrag, already at lower dosage, besides its prokinetic activity, has a beneficial effect on colonic and serum inflammation.

### αS accumulation in the colon of A53T Tg mice appears not affected by Velusetrag treatment

**Fig. 6:**
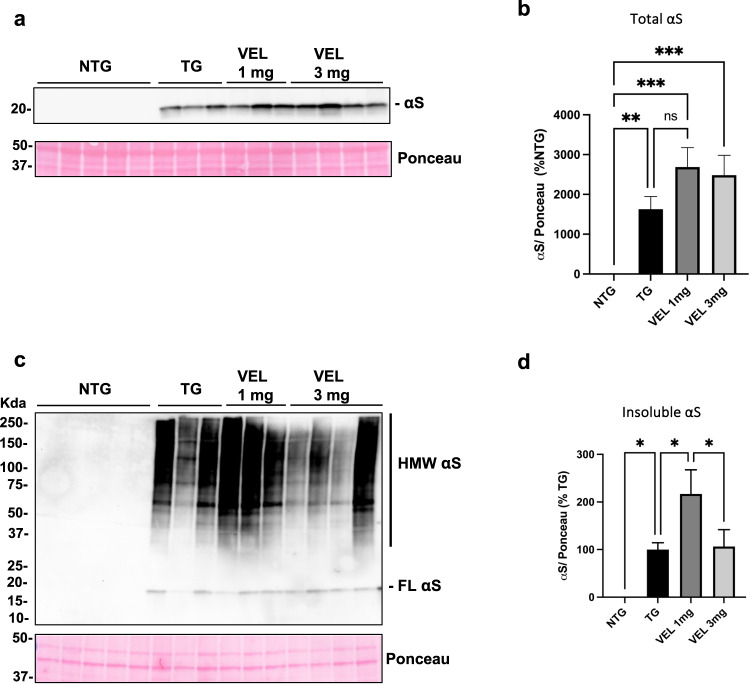
αS expression and aggregation appears not affected by the administration of Velusetrag. Expression level and aggregation of αS was evaluated in Tg mice and littermates after the treatment with Velusetrag. **a**, **b** Total fractions from distal colon sections of treated mice were evaluated through WB with Syn1 antibody. Protein bands intensity was determined using ImageLab and are normalized with the total protein load visualized through Ponceau staining. **b** Graph of protein analysis shows no significant changes in αS total expression level. Values are expressed as % of Ntg and given as the mean ± SEM (*n* = 4–5, **p < 0.01; ***p < 0.001; one-way ANOVA, Fisher’s LSD post hoc test). **c**, **d** Detergent-insoluble fractions obtained from the distal colon of treated Tg and Ntg littermates were blotted with Syn1 antibodies to evaluate possible changes in αS aggregation. Intensity of insoluble αS species was determined using ImageLab and are normalized with the total protein load visualized through Ponceau staining. HMW = high molecular weight **d** Graph of protein analysis of insoluble αS species. Values are expressed as % of Tg and given as the mean ± SEM (*n* = 4–5, (*p < 0.05, **p < 0.01; one-way ANOVA, Fisher’s LSD post hoc test).

Since our evidence showed a strong impact of Velusetrag on colonic function and gut and peripheral inflammation in the A53T mice, we also examined the action of the 5-HT4 agonist Velusetrag on αS expression and aggregation. (Fig. 6a–d). Besides an overall variability in αS protein level and aggregation within the same group, it appeared that the overexpression and the aggregation of the protein did not statically change after Velusetrag treatment. The amount of αS aggregates, evaluated in the detergent-insoluble fraction seemed to be increased in mice treated with the 1 mg dose, whereas the 3 mgs did not. Such high variability within groups for αS aggregates in the colon of this line at 6 months is somehow expected since the level of aggregation may depend on the time of onset of the motor phenotype and on the aggressiveness of its progression, which may differ between Tg mice, especially in the prodromal phase.

### Microbiome analysis reveals that Velusetrag treatment re-established normal gut microbial communities in the A53T line

GI dysfunction is often accompanied by gut dysbiosis in PD patients, a phenomenon that has been described also for Tg animal models of alpha-synucleinopathy^36,37^. Interestingly, the αS A53T line showed a constant alteration in the level of small chain fatty acids at an early age, suggesting possible abnormalities in the GI microbial resident population^35^. Therefore, we analyzed fecal pellet composition in all groups of animals, including those treated with Velusetrag, to evaluate how changes in constipation due to the treatment with this 5-HT4 agonist impacted the gut microbiome.

**Fig. 7:**
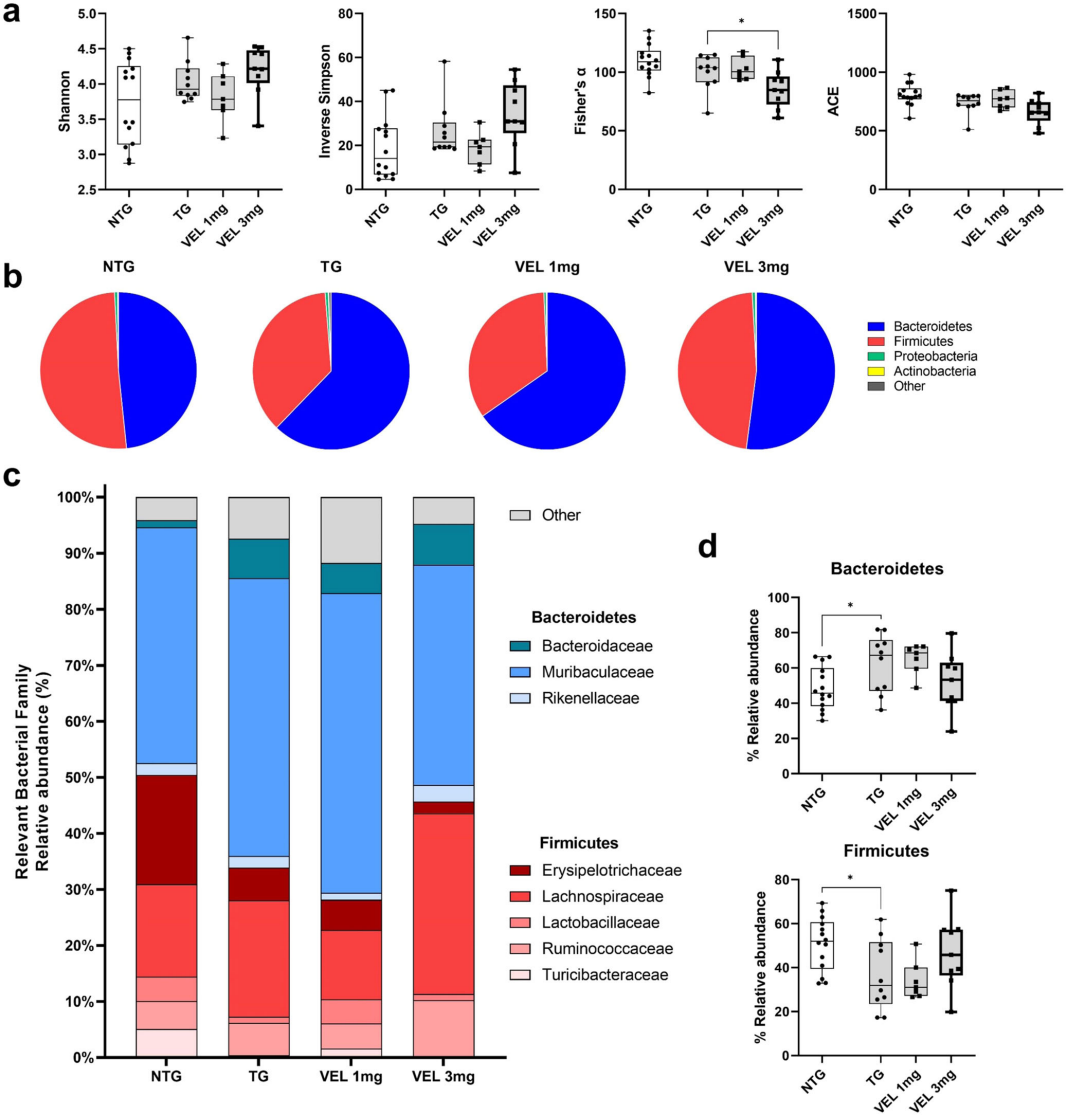
Microbiome analysis reveals that Velusetrag treatment re-established normal gut microbial communities in the A53T line. Differences in α-diversity, β-diversity, and the microbiota composition at the phyla and family level of the four groups were represented. **a** α-diversity of gut microbiota was assessed in animals treated with Velusetrag or water and Ntg littermates by Shannon, Simpson, Fisher and ACE indices. **b** Pie-chart of the top four dominant phyla of gut microbiota in each treatment group. **c** Comparison of relative abundances of different dominant phyla in distinct treatment groups. **d** Relative abundance of 8 most relevant bacterial families of each sample in the different treatment groups. **a**–**d** The between-treatment group comparisons were statistically assessed with T-test (Ntg vs Tg mice) or Kruskal–Wallis followed by the Dunnet’s test (VEL 1 mg or VEL 3 mg vs Tg control mice) (*p < 0.05). **a**, **d** In each box plot, the center line corresponds to the median value and the bound of box corresponds to the 25^th^ and 75^th^ percentile, respectively. All points are shown and whiskers are plotted down to the smallest value and up to the largest one.

The within-sample diversity (aka α-diversity) was assessed using four associated indices. According to the results of α-diversity no statistical significant difference between Ntg and Tg samples was found and, also comparing Tg animals with VEL 1 mg mice, values were found quite similar. VEL 3 mg samples were higher compared with Tg mice according to Shannon and Inverse Simpson indices while, in Fisher’s and ACE indices (Fig. 7a), the highest results were obtained in Ntg animals and the lowest were found in VEL 3 mg group.

The following analysis of bacterial composition confirmed this initial observation, showing that the αS A53T mice at 6 months of age presented a significant dysbiosis compared to age-matched Ntg littermates (Fig. 7b). Ntgs had a well-balanced amount of *Firmicutes* and *Bacteroidetes* phyla but this balance was importantly affected in Tg animals. Specifically, these changes seen in Tg mice accounted for an increase in *Bacteroidetes* with a concomitant reduction in *Firmicutes* representatives. Remarkably, the administration of Velusetrag at the highest dosage, normalized bacterial composition, by restabilising a microbial pattern more similar to Ntg littermates than to Tgs treated only with vehicle. While the microbiome composition in Velusetrag 1 mg group was even more unbalanced than in Tg mice only treated with water, the group of 3 mg Velusetrag recovered completely the condition shown in Ntg animals, with a concomitant increase of *Firmicutes* and the corresponding reduction of *Bacteroidetes*.

Among the dominant phyla, only *Bacteroidetes* and *Firmicutes* showed statistically significant differences. *Actinobacteria* and *Proteobacteria* were equally represented in all the treatment groups while, surprisingly, *Verrucomicrobia* were absent in all the samples (Fig. 7c). Further investigations to identify differentially abundant families within the groups, confirmed that the changes of *Bacteroidetes* phylum seen after 3 mg Velusetrag were represented mainly by the decrease of *Muribaculaceae* and the increase of *Bacteroidaceae* families, where the latter were mostly correlated to the Tg phenotype of the mice than to the application of the Velusetrag treatment.

The treatment with 3 mg Velusetrag influenced also other families, depleting *Erysipelotrichaceae* and supporting the increase of *Lachnospiraceae* and *Ruminococcaceae* showing a progression compared to other Tg animals and the ability to stimulate a substitution within *Firmicutes* phylum together with the above-mentioned reduction of *Bacteroidetes* (Fig. 7d).

## Discussion

5-HT has been recognized for decades as an important signaling molecule in the gut. The GI tract has five of the seven 5-HT receptor subtypes, 5-HT1, 5-HT2, 5-HT3, 5-HT4, and 5-HT7^17^. Among these, the 5-HT3 and 5-HT4 receptors are involved in the gut propulsive motility and in the neurogenic secretory responses. Therefore, modulation of 5-HT pathway can be a successful treatment for GI disorders. For instance, inhibition of the 5-HT3 receptor activates descending reflex pathways that affect circular muscle activity^19^. The result is a drastic reduction of peristalsis caused by the termination of the ascending contractile reflexes. Because of this effect, 5-HT3 receptor antagonists have been used to treat diarrhea and as antiemetic drugs during chemotherapy. Conversely, the 5-HT4 receptor is selectively implicated in stimulating gut motility to counteract constipation^21^. Over the years, several molecules agonist of the 5-HT4 receptor have been developed and tested for the treatment of chronic constipation. However, their use had been restricted due to their severe side effects on the cardiac apparatus. Myocardial infarction, unstable angina, or stroke were the side effects of Tegaserod, likely due to its interaction with non-5-HT4 receptors^38^. Other prokinetics of the old generation, such as cisapride, domperidone, and metoclopramide, also interact with multiple non-5-HT4 receptors (i.e., 5-HT2A, 5-HT2B, or 5-HT3) or hERG (human *ether-a-go-go*-related gene) K^+^ channels, increasing the risk of cardiovascular side effects^39^. It appeared clear that, to cure chronic constipation, avoiding severe adverse effects became a goal to be pursued. Therefore, many efforts were made to develop molecules with as much affinity as possible for the 5-HT4 receptor. At present, two compounds have entered clinical studies as highly selective 5-HT4 receptor agonists. One is Prucalopride (Resolor; Movetis NV, Turnhout, Belgium), which was approved by the EMA for clinical use in 2009 and marketed in some European countries like Germany and the UK and has been used to treat diabetic constipation with no risk for the cardiovascular system^40,41^. The other highly selective 5-HT4 receptor agonist is Velusetrag. Velusetrag has shown no appreciable affinity for the other serotonin receptor subtypes and phase 2 studies demonstrated how this drug improves symptoms and the quality of life of chronically constipated subjects after a single daily administration for 4 weeks^22–24^. Symptomatic improvement in patients with diabetic or idiopathic gastroparesis was also observed already at 5 mg of dosage after 12 weeks treatment period^24^.

In this study, we have extensively investigated the effect of Velusetrag on the colonic deficit of a Tg mouse model of PD with severe constipation. We found that a month of Velusetrag regimen was well-tolerated and improved GI motility, reduced peripheral and colonic inflammation, improved axonal degeneration and ultimately re-established a normal, well-balanced microbiome. The PD mouse model used, the PrP human A53T αS Tg mice, is characterized by an early onset of GI dysfunctions that include slower colonic motility, reduced stool frequency, and abnormally elongated stools^27^. Such deficit is linked to a reduced cholinergic transmission with concomitant accumulation of aggregated αS in the colon that takes place at least 6 months before accumulation of inclusions and neurodegeneration in the CNS.

We initially hypothesized that the cholinergic abnormalities in the large intestine could be the result of a local, selective loss of specific neuronal populations as it has been postulated in patients although a conclusive demonstration is still missing^42^. As shown in this study, no neuronal loss including ChAT^+^ neurons were found in the distal colon of Tg mice. Instead, the real surprising finding was to observe axonal degeneration, particularly of cholinergic innervation, as shown by reduced staining with NF-H and VAChT. Such a decrease could indeed explain the deficit in synaptic transmission, seen in this model through ex-vivo measurements. Notably, alteration of cholinergic innervation in the colon of PD patients has been recently described^14,15^ and αS was found to colocalize with cholinergic neurons and possibly regulate their function^27,31^. Unexpectedly total level of colonic ACh was not significantly changed in 6-month-old Tg mice. Since total colonic ACh was measured in this study and not the specific pool of ACh secreted during contractions, further and more precise investigation, such as in vivo recording of ACh release, will be necessary to fully evaluate this aspect. In line with this, it would be also important to further elucidate DA contribution to intestinal motility in the PrP αS Tg line and its modulation with Velusetrag. Unexpectedly, TH expression level was found to increase in the distal colon of Tg mice treated only with vehicle, compared to Ntg littermates, an aspect completely counterintuitive for a PD model but that could be also linked to sympathetic denervation^43^. Notably, according to early data, nigrostratial degeneration of dopaminergic neurons in this mouse model, as well as striatal DA level, did not significantly change, even in concomitance of sustained, brain α-synucleinopathy^44^, although alterations in the level of the DA transporter (DAT) in the striatum and the D1 receptor in the substantia nigra in presymptomatic 9 months old mice were later described^45^. Because of this apparent lack of dopaminergic dysfunction in the brain, this PrP A53T Tg line was often more appreciated as a model of αS pathology rather than a system to study PD-related dopaminergic neuronal loss. This new evidence of a TH overexpression in the distal colon, together with the above-mentioned changes in DAT and D1 receptor in the CNS of this Tg line in a presymptomatic stage, could potentially challenge this view. At the same time, the role of DA in the gut has proven difficult to elucidate in humans and animal models. Recently, single nuclei sequencing data have shown the existence of specific neuronal population expressing TH and dopamine-β-hydroxylase in the mouse colon^11,12^, whereas evidence obtained from ex-vivo stimulation of colonic preparations demonstrated that DA can modulate GI motility^16^. In addition, the expression of all 5 classes of DA receptors have been described in the intestine^46^. Therefore, increased TH expression in the GI tract of Tg mice should be further investigated, in connection to the production of DA and other catecholaminergic neurotransmitters, to better define its contribution to colon motility and to the inhibition of cholinergic transmission.

No significant change was found in the level of αS expression or αS aggregation after Velusetrag treatment, suggesting that this compound may not directly affect αS biology and its tendency to aggregate. Instead, the administration of Velusetrag had a profound impact on constipation in the A53T mice. Not only the treatment with this 5-HT4 agonist improved colonic motility in the short term but also induced long lasting effects on the axonal network in the ENS of the large intestine, as seen by the recovery of signal for NF-H (*p* = 0.06 for 3 mg compared to Tg vehicle), for VAChT as well as a normalization in the level of TH.

Notably, pro-regenerative effect on ENS circuits has been reported for other 5-HT4 agonists. For example, in guinea pigs continuous, local infusion of mosapride, a first generation 5-HT4 agonist, after rectal transection, increased neuronal numbers and length of neurites of the neural circuit in the impaired myenteric plexus and the recovery of the defecation reflex in the distal gut^28^. More data will be necessary to confirm a pro-regenerative effect of Velusetrag on the ENS network.

Moreover, peripheral, and colonic inflammation was selectively counteracted by Velusetrag treatment. Increase of proinflammatory cytokines, alteration of the intestinal epithelial barrier with elevated permeability, and elevated number of intestinal glia cells are a salient feature of the A53T line and also found in PD patients^35,42^. Velusetrag regimen at both dosages was particularly successful in reducing cytokines levels (IL1β and TNF-α) both in plasma and in colon, but also in counteracting colonic glia activation at lower dosage. The 3 mg dose of Velusetrag was not particularly effective on glia engagement, an observation that could be related to initial desensitization of the 5-HT4 receptor and would need further investigation. While this would be the initial evidence for an anti-inflammatory effect of Velusetrag, the other 5-HT4 agonist available, Prucalopride, has been described to reduce proinflammatory cytokines expression and the influx of inflammatory cells in the intestine after abdominal surgery in mice and in humans^47^.

Reduction of intestinal inflammation could also be observed by the microbiome shift in Tg mice treated with Velusetrag. Notably, the A53T mice show alteration in the level of butyrate and propionate from an early age^35^. Analysis of the S16 DNA sequence in 6-month-old mice showed overall an unbalanced microbiota composition with a reduction of the *Firmicutes* phylum and the corresponding increase in *Bacteroidetes*. Velusetrag treatment at the highest dose employed in this study, normalized the microbiota, by reestablishing a well-balanced microbiome similar to control Ntg mice. On the opposite, the 1 mg dose of Velusetrag did not affect the microbial population of Tg mice, which was found comparable to untreated Tgs. When the presence of relevant bacterial families was analyzed more in detail some of the dysbiosis traits observed in PD patients could also be seen in untreated A53T mice. For example, other authors^48–50^, have reported that *Prevotellaceae* were differentially abundant between PD and control subjects. Surprisingly, in our study *Prevotellaceae* family was found positively enriched in Tg and VEL 1 mg samples but detected in extremely low amounts after Velusetrag administration, a condition comparable to the Ntg group.

Interesting fluctuations were found in some of the most relevant families as *Erysipelotrichaceae*, *Lachnospiraceae* and *Ruminococcaceae*, as correlated to inflammation or SCFA production, respectively: *Erysipelotrichaceae*, frequently reported as enriched in proinflammatory genera^51,52^, were strongly represented in Ntg animals but progressively reduced in Tg and VEL 1 mg groups, being almost completely depleted in VEL 3 mg animals. On the opposite, SCFA producers as *Lachnospiraceae* and *Ruminococcaceae* families, members of *Firmicutes* phylum, were strongly enriched in VEL 3 mg, filling the gap left by the reduction of *Muribaculaceae*/*Bacteroidetes* and substituting *Erysipelotrichaceae*, with a further contribution towards anti-inflammatory effect.

Many authors have highlighted the increased abundance of *Lactobacillus* genus in stools of PD subjects^52–56^ and its increase was reported as an indication of gut dysbiosis in terms of significant alterations of the normal composition of human and mice microbiota. Furthermore, in an interesting study by van Kessel et al. ^57^, the increase of *Lactobacillus* showed significant negative correlation between species from the genus *Lactobacillus* and levodopa uptake. Our results showed that, in control animals (Ntg), *Lactobacillaceae* amount was quite high and, differently from literature, the disease model induced a reduction of this family. In VEL 1 mg *Lactobacillaceae* levels were similar to Ntg animals but the administration of VEL 3 mg significantly counteracted such increase.

Finally*, Turicibacteraceae* were found significantly increased in the microbiota of PD patients and were correlated with disease severity, medication, and non-motor symptoms^58^. Our results showed that *Turicibacteraceae* were enriched in Ntg samples and, to a lower extent, in VEL 1 mg, while Tg animals presented a significant depletion of the family which was almost complete only in VEL 3 mg group.

Overall Velusetrag treatment rescued GI dysfunction, gut inflammation, and dysbiosis in the A53T mice. No well-defined dose-dependent effect was found for all the read outs, suggesting that maybe a non-optimal dosage was used, especially for the 3 mg regimen. When addressing drug-receptor binding, it is also important to bear in mind that the magnitude of the response to a given drug concentration can fluctuate widely between different individuals, and that the achievement of a certain percentage value of maximum effect can come within an even very wide spectrum of drug concentrations. Biological variability is therefore a concept that must always be kept in mind during the course of a pharmacological treatment, both in terms of magnitude of response and pharmacokinetics. The use of a larger number of animals would perhaps have led to an optimal dose-response curve.

Nevertheless, Velusetrag had a remarkable effect on GI dysfunction in the A53T mice with no side effects, making this drug a promising candidate to treat chronic constipation in PD.

## Methods

### Ethics & inclusion statement

This research was the result of a collaboration between Scuola Normale Superiore and the pharmaceutical company Alfasigma. All local researchers were included in the study implementation, data ownership, intellectual property, and authorship of this publication. Roles and responsibilities for each researcher were agreed amongst collaborators ahead of the research. This research was not subject to local restrictions and the use of animals in research was authorized by the Italian Ministry of Health as indicated in the manuscript.

### Mice

Line G2–3 (Prnp-SNCA*A53T)23Mkle/J) is a Tg mouse line expressing human A53T αS under the control of the mouse prion protein (PrP) promoter^44^. Appearance of motor symptoms that include reduction in ambulation, wobbling, lack of balance, and weakness of the hind limbs occurs in these mice since 9 months of age and is associated with neurodegeneration, neuroinflammation, and typical αS pathology in the CNS. Non-motor symptoms such as severe constipation are also common in this model but manifest much earlier. The intestinal dysfunction in this line includes delayed GI transit time, abnormal colon contractility associated with a defect in cholinergic transmission, colonic inflammation, impaired permeability of the intestinal barrier. All GI symptoms are prodromal to the CNS dysfunction, already present at 3 months of age in concomitance to the accumulation of colonic αS inclusions^27,35^. For this study, litters were obtained by crossing a Tg male x C57Bl/6 J female and were genotyped after 3 weeks from birth. αS transgene carriers were labeled as Tg, whereas non-carrier individuals were non-Tg (Ntg) and were used as controls. Presymptomatic Tg mice at 5 months of age and age-matched Ntg littermates were used. Mice were housed under standard conditions with a 12 h light/dark cycle with free access to food and water. All animal studies were approved by and complied in full with the Italian and European laws for laboratory animal welfare and experimentation (Directive 2010/63/EU and Italian Legislative Decree 26/2014) and the experimental protocol was approved by the Italian Ministry of Health (Authorization no. 749/2021-PR).

### Pharmacological treatment

Velusetrag was prepared fresh each time before use and dissolved in either 3 mg/10 ml sterile water/kg or 1 mg/10 ml sterile water/kg. The two doses were selected on the basis of those administered to patients (15 mg/daily e 5 mg/daily) and calculated according to the table of species interconversion of FDA (U.S. Department of Health and Human Services, Food and Drug Administration, Center for Drug Evaluation and Research (CDER) July 2005, Pharmacology and Toxicology). A total amount of 45 Tg mice were divided into three groups: 15 mice were treated with 3 mg/10 ml sterile water/kg, 15 mice with 1 mg/10 ml sterile water/kg, and a group of 15 Tg mice were treated with sterile water (vehicle). The control group of 15 Ntg mice was treated with vehicle. Mice were chosen randomly for each treatment. Both male and female animals were used. The treatment was administered by oral gavage daily for 28 days. The groups of mice were all monitored two times weekly for signs of changes in body weight or motor dysfunction. Before tissue collection, mice were evaluated with behavioral tests, and stools were collected for microbiome analysis.

### Behavioral tests

#### Body weight

All mice were weighed twice/week to assess any weight loss or gain following the drug treatment. Weight was recorded.

#### Gait test

The gait test was performed as described in Rota et al.^27^, Girirajan et al.^59^, and Wertman et al.^60^. The gait test was assessed in mice 3 weeks after starting the treatment with either Velusetrag or vehicle. Each animal was tested once on a particular test day. The forepaws of each mouse were painted with washable, edible blue paint and the hind paws with red dye. Every animal was placed to walk alongside a strip of white paper into a linear maze, at the end of which an edible chow was inserted and the footprints were let dry. For each mouse 4 footprints, corresponding to a walking step, were selected and stride length (distance between anterior and posterior same-side footprints), sway length (distance between anterior or posterior footprints) and stance length (distance between anterior left and posterior right footprint or vice versa) were measured and recorded.

#### Stool collection

The stool collection was performed before the sacrifice of mice treated with either Velusetrag or a vehicle. Each animal was placed alone into a clean plastic cage with no access to food and water. Pellets were collected and placed in individual Eppendorf tubes. The stool weight was measured fresh and after drying at 65 °C o/n to determine the water content, which was calculated as a difference between total weight and dry weight.

### Contractile activity of colonic longitudinal and circular smooth muscle

The colon was removed and placed in cold Krebs solution to record contractile activity, using an isometric force transducer. As previously described^61^, after sacrifice, longitudinal and circular muscle strips of the intestine were allocated in organ baths containing Krebs solution at 37 °C, bubbled with 95% O_2_ + 5% CO_2_. The strips were connected to an isometric force transducer and the mechanical activity was recorded as a measure of tension using a BIOPAC MP150 system. A pair of coaxial platinum electrodes were positioned at a distance of 10 mm from the longitudinal axis of each preparation to deliver transmural electrical stimulation (ES) by a BM-ST6 stimulator. ES was applied as 10-s single trains consisting of square wave pulses (0.5 ms, 30 mA). Muscle contractions were recorded upon stimulation and the tension developed by each strip (grams) was normalized by the weight of the corresponding wet tissue (g/g tissue).

### Evaluation of colonic and plasma cytokines and colonic ACh

The evaluation of TNF-α and IL1β levels in colonic and plasma tissues was performed by ELISA kits (for TNF-α, Thermofisher, Waltham, MA, USA and for IL1β, Abcam, Cambridge, UK)^35^. Colonic tissues were homogenized in PBS, pH 7.2 at 4 °C, then centrifuged at 10,000 × *g* for 5 min. Aliquots of supernatants were diluted 1:4 with the diluent buffer provided and used for each test. The concentrations of TNF-α and IL1β, corrected by the dilution factor, were expressed as picograms per milligram of tissue. Plasma tissue was diluted 1:4 with the diluent buffer provided and used for each test. The concentrations, corrected by the dilution factor, were expressed as picograms per milliliter.

The evaluation of ACh levels in colonic tissues was performed by ELISA kit (Abcam)^62^. Colonic tissue was homogenized in choline assay buffer provided with the kit, at 4 °C, then centrifuged at 10,000 × *g* for 5 min. The concentration of ACh was expressed as nmol per milligram of tissue.

### Western blotting analysis

For western blotting (WB) analysis, the mouse colon was divided into a proximal and distal segment, flushed of fecal content, frozen on dry ice, and stored at −80 °C until use. For our aims, we used only the distal portion of each colon. Preparations of colonic lysates were performed as described in Rota et al., 2019^27^. Briefly, frozen tissues were homogenized using a Potter-Elvehjem Grinder homogenizer on ice in 20% (w/v) TNE lysis buffer (50 mM Tris-HCl pH 7.4, 100 mM NaCl, 0.1 mM EDTA) with proteases and phosphatases inhibitors. 100 μl of homogenate was processed to obtain total lysates by adding an equal volume of TNE buffer containing 2% NP-40, 2% SDS, 0.2% DOC. Total lysates were then sonicated and boiled at 95 °C. 400 μl of original homogenate was processed to obtain detergent-soluble and insoluble fractions. Briefly, an equal volume of TNE buffer containing 2% of NP-40 was added. The homogenates were then centrifuged at 10,000 × *g* at 4 °C to collect NP-40 soluble and insoluble fractions. Pellets were then washed one time with TNE buffer with 1% of NP-40 and resuspended in half of the original volume in TNE containing 1% NP-40, 1% SDS, 0.1% DOC. NP-40 insoluble fractions were then sonicated and boiled for 5 min at 95 °C. Protein amounts were determined with BCA assay (Euroclone, Milan, Italy). Tissue lysates were run on a 4–20% Criterion™ TGX™ Precast Midi Protein Gel (Bio-Rad, Hercules, CA, USA) and afterwards transferred onto nitrocellulose membrane at 200 mA, o/n at 4 °C, using carbonate transfer buffer^63^. The membranes were blocked with 5% non-fat dry milk in 1× PBS containing 0.01% Tween-20 (PBS-T) at RT for 30 min. Membranes were then incubated with the specific primary antibody, properly diluted in 2.5% non-fat dry milk in PBS-T, o/n at 4 °C. The following primary antibodies were used: 5-Hydroxytryptamine receptor 4 (5-HT4R) (Bioss Antibodies, MA); phospho-AKT, AKT, phospho-mTOR and mTOR (Cell Signaling, MA, USA); Syn1 antibody (BD Biosciences, NJ, USA). The day after, membranes were washed with PBS-T and incubated for 1 hr at RT with the matching horseradish peroxidase-conjugated secondary antibody in 2.5% non-fat milk PBS-T solution. The chemiluminescent signal was visualized using a CCD-based Bio-Rad Molecular Imager ChemiDoc System (Bio-Rad). Band intensity was quantified using ImageLab software (Bio-Rad) and normalized for the relative intensity of the Ponceau staining.

All blots or gels were derived from the same experiment, and they were processed in parallel.

### Whole mount staining

For immunohistochemical analysis whole mount colon preparations were performed as described by Gries M. and colleagues^64^. Mouse intestine was removed, and the segment was opened longitudinally along the mesenteric border, laid with the mucosa side up, stretched flat, and fixed by thin needles on a wafer-thin balsa wood in a petri dish. Fixation was done with 4% paraformaldehyde (PFA) for 24 h at 4 °C. The day after, samples were washed three times with PBS for 30 min. Intestinal wall samples were cut into 8 sections of approximately equal length and subsequently, each section was divided into 4 subsections, as shown in supplementary Fig. 2a, b, and stored in 0.1% NaN_3_/PBS at 4 °C. For immunostaining subsections from sections 1, 2, and 3 of the distal colon were used. Tissues were permeabilized for 4 h at 37 °C on a shaker with a solution containing 0.01% NaN_3_, 1% normal goat serum (NGS, Cell Signaling), 1% Triton-X-100 in PBS. Then, samples were blocked in blocking solution (0.01% NaN_3_, 10% NGS, 0.1% BSA, 1% Triton-X100 in PBS) o/n at 4 °C on a shaker. Subsequently, samples were incubated with primary antibodies, diluted in blocking solution, at 37 °C for 48 h on an orbital shaker. After rinsing four times in PBS-T (0.05% Tween-20 in PBS) for 30 min, tissues were incubated with respective Alexa Fluor secondary antibodies (Thermofisher) in PBS containing 0.01% NaN_3_ and 1% Triton-X-100 for o/n at 37 °C on an orbital shaker. Again, samples were rinsed with PBS-T four times for 30 min and then counterstained with DAPI (1:1000) for 2 h at RT. Samples were washed in PBS four times for 30 min at RT and finally mounted on a Super-Frost Plus glass slide using Fluormount (Sigma Aldrich, St. Louis, Missouri USA). The following primary antibodies were used: glial fibrillary acidic protein (GFAP, Thermofisher), PGP9.5, tyrosine hydroxylase (TH, Abcam), neurofilament H (NF-H), choline acetyltransferase (ChAT) and vesicular acetylcholine transporter (VAChT, Millipore, Burlington, MA, USA).

For IF analysis, 10–15 images of whole mount distal colon preparations (2.5 mm^2^) from three different animals for each group, were analyzed (*n* = 10–15/group). Z-stacked images were acquired with a Zeiss LSM 900 airyscan 2 confocal microscope, using a Plan-Apochromat 40×/01.4 oil DIC (UV) VIS-IR M27 objective (Carl Zeiss Microscopy GmbH). Images were visualized with the Zeiss ZEN 3.1 (blue edition) software. All images were captured at identical laser strength and gain conditions that were selected for each antibody signal. For image analysis, in the case of GFAP, Pgp9.5, ChAT, and DAPI staining, the number of cell bodies and nuclei were manually counted using the Fiji Cell Counter plugin. GFAP, Pgp9.5, and ChAT-positive glial or neuronal cell bodies were identified in areas where the nucleus and cytoplasm were clearly visualized. For analysis of NF-H, VAChT, or TH staining, for each image maximum projection of each z-series was obtained and the mean fluorescence intensity was quantified following the approach 2—“threshold method” from the protocol by Shihan et al.^65^. Examples of this approach are visible in Suppl. Fig. 2d. Briefly, from the Adjust function of the Fiji software threshold values were selected based on the nature of the image and fluorescence intensity. The wand tracing tool was then used for the selection of the region of interest and selected areas were measured from the Analyze function.

### Statistical analysis

All data are presented as the mean ± SEM. All measurements were taken from different samples, except for immunostaining where 3–5 images were taken from the same section. Differences between means were evaluated by One-way ANOVA (Prism, Graph Pad Software, San Diego, CA, USA). Post hoc tests were applied for multiple comparisons between Tg-vehicle as control and all the other groups.

### Microbiome analysis

During the stool collection, a few pellets from each mouse were collected and placed in separate Eppendorf tubes and immediately frozen in dry ice for microbiome analysis.

Fecal bacterial DNA was extracted using the FastDNA SPIN Kit for soil and FastPrep Instrument (MP Biomedicals, Santa Ana, CA, USA). The extracted DNA was quantified using the picogreen method of the Quant-iT™ HS ds-DNA assay kit in a Qubit™ fluorometer (Thermofisher) and verified. DNA extracts were diluted to a concentration of 10 ng/μl for reducing the template amount associated PCR biases.

The V4–5 hypervariable regions of the bacterial 16 S rRNA gene were amplified and sequenced by the Integrated Microbiome Resource institute (Dalhousie University, Halifax, Canada) to obtain the microbial composition of the analyzed samples. Amplicon libraries were generated with primers based on the 515FB (5′-GTGYCAGCMGCCGCGGTAA-3′) /926 R (5′-CCGYCAATTYMTTTRAGTTT-3′) as suggested previously^66^. The sequencing instrumentation, methodology, and chemistry were based on the Illumina MiSeq instrument using the 2 × 300 bp paired-end v3 chemistry as detailed by Comeau et al. ^67^. The sequences were quality trimmed with Trimmomatic v0.39 using the default settings (with the exception if 5′ trimming) and a minimum length cutoff of 100 bp after the sample index trimming (the average amplicon length was 410 bp)^68^. Total amplicon sequences were reconstructed through assembly of the read pairs with the FLASH v1.2.11 software using the default parameters^69^. Further, sequence screening for sequencing errors and PCR-introduced chimeras, alignment to reference databases, and generation of OTU matrices were performed with the Mothur v1.45.2 software suit^70^. Chimeric amplicons were identified and removed using the abundance-based de novo UCHIME v4.2.40 approach^71^. A further classification step, using the naïve Bayesian classifier as implemented in Mothur, was performed for identifying and further removing sequences classified in non-target taxa (unknown, eukaryotic, chloroplasts, and mitochondria)^72^. Sequence distances were calculated for the remaining aligned sequences, while clustering of sequences into 0.03 distance-defined operational taxonomic units (OTUs) was performed with USEARCH^73^.

Sequencing effort coverage and α-diversity indices were calculated with the entropart v1.6.6^74^ and the vegan v2.5.7^75^ packages of the R software^76^ with vegan being used also for the multivariate approaches. The Good’s coverage estimate for assessing sequencing depth, along with measures of α-diversity like the Shannon index, the reciprocal Simpson index, the Fisher’s α index, and ACE richness estimator were calculated. ANOVA and a Tukey’s post hoc analysis or the non-parametric equivalents of the Kruskal–Wallis test followed by the Fisher’s LSD test were implemented for assessing statistically significant differences between the treatment groups and time-points for the α-diversity indices, using the Agricolae v1.3.5 package of the R software^77^. The vegan package was also used to perform between sample analysis (β-diversity analysis).

A nonmetric multidimensional scaling analysis (nMDA) was performed to identify non-parametric sample compositional OTU relations. Redundancy analysis (RDA) was used, for testing the effects of the treatments compared with all control groups. The non-parametric Kruskal–Wallis test followed by the Fisher’s LSD test (if the Kruskal–Wallis test was significant) was used for identifying differentially abundant OTUs among the different treatments and genotypes in order to assess the potential effect at an OTU level as previously suggested^78^.

Prism software was used for statistical analyses. Results were indicated as the median with min to max points. In detail, all the parameters were evaluated applying Student’s T-test (parametric or non-parametric according to normal distribution of data) comparing Ntg vs Tg (*p < 0.05) followed by One-Way ANOVA (or the corresponding non-parametric Kruskal–Wallis) with Dunnett’s multiple comparison test (or Dunn’s multiple comparisons test) for Tg vs different dosage of VEL (1 mg and 3 mg) (*p < 0.05).

#### Sequencing data quality control

The sequencing raw reads were subjected to a series of quality control steps for the removal of PCR and sequencing artifacts, and this resulted in the reduction of the sequence dataset by ~48.07% (from 3,303,120 to 1,715,372 sequences). The remaining sequences showed an achieved coverage of 99.6 ± 0.11% of the existing bacterial diversity among OTUs of ≥0.1% in relative abundance according to the Good’s coverage estimate.

### Reporting summary

Further information on research design is available in the Nature Research Reporting Summary linked to this article.

### Supplementary information

Supplemental figures

Reporting summary

## Data Availability

The data that support the findings of this study are available from the corresponding authors upon request. Biological material can be obtained from the corresponding authors upon request.
