# Supplementary material for: Velusetrag rescues GI dysfunction, gut inflammation and dysbiosis in a mouse model of Parkinson’s disease
Source: NPJ Parkinsons Dis. 2023 Oct 2;9:140. doi: 10.1038/s41531-023-00582-1 (PMC10545757; doi:10.1038/s41531-023-00582-1)
Supplement: Supplementary file 1 — Supplemental figures [file 41531_2023_582_MOESM1_ESM.pdf]

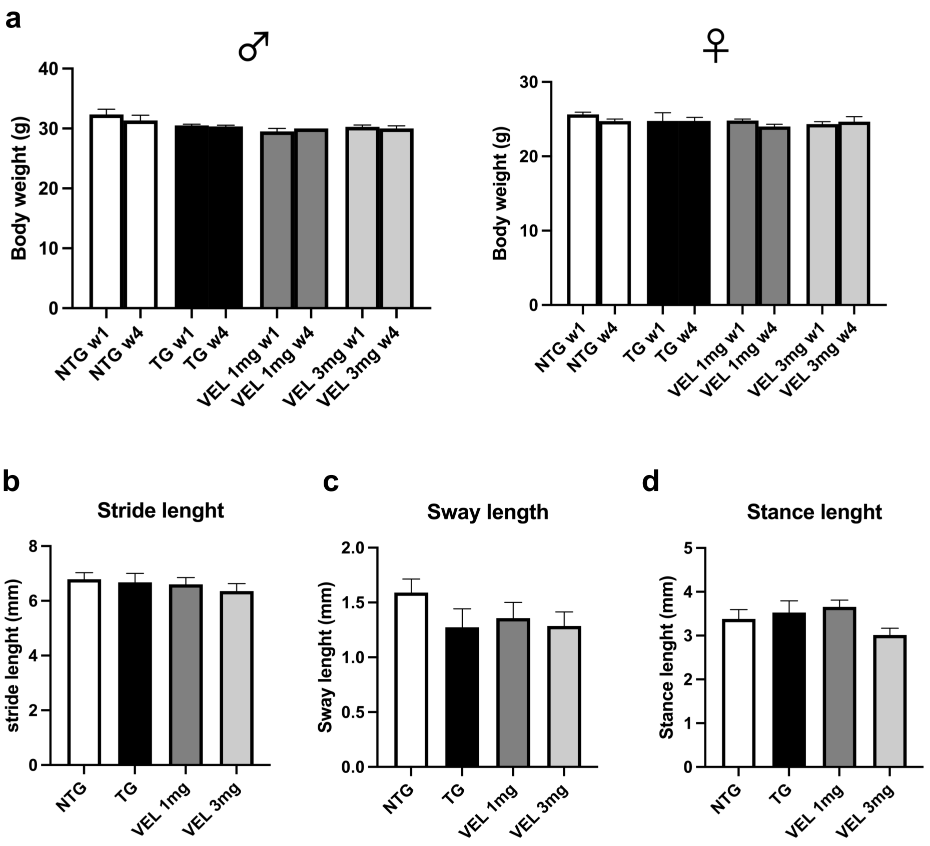

Supplementary Fig. 1 Velusetrag treatment is well-tolerated by the A53T Tg line.

Body weight and motor coordination was determined in mice treated either with Velusetrag or vehicle. **a** Mice were weighed twice each week. The graphs show the mean body weight at week 1 (w1) and week 4 (w4), divided by gender. Values are given as the mean ± SEM (n=10-14, One-way ANOVA, Tukey post hoc test). **b,c,d** Gait test was assayed in Velusetrag treated animals and controls after 3 weeks of treatment. Mice with painted paws were induced to walk on paper placed into a maze and distances between strides (b), sway (c) and stance (d) was recorded. As expected, no significant difference was found in motor activity and balance at this age. Values on graphs are expressed as raw data and are given as the mean ± SEM (n= 10-14, one-way ANOVA followed by Tukey post hoc test).

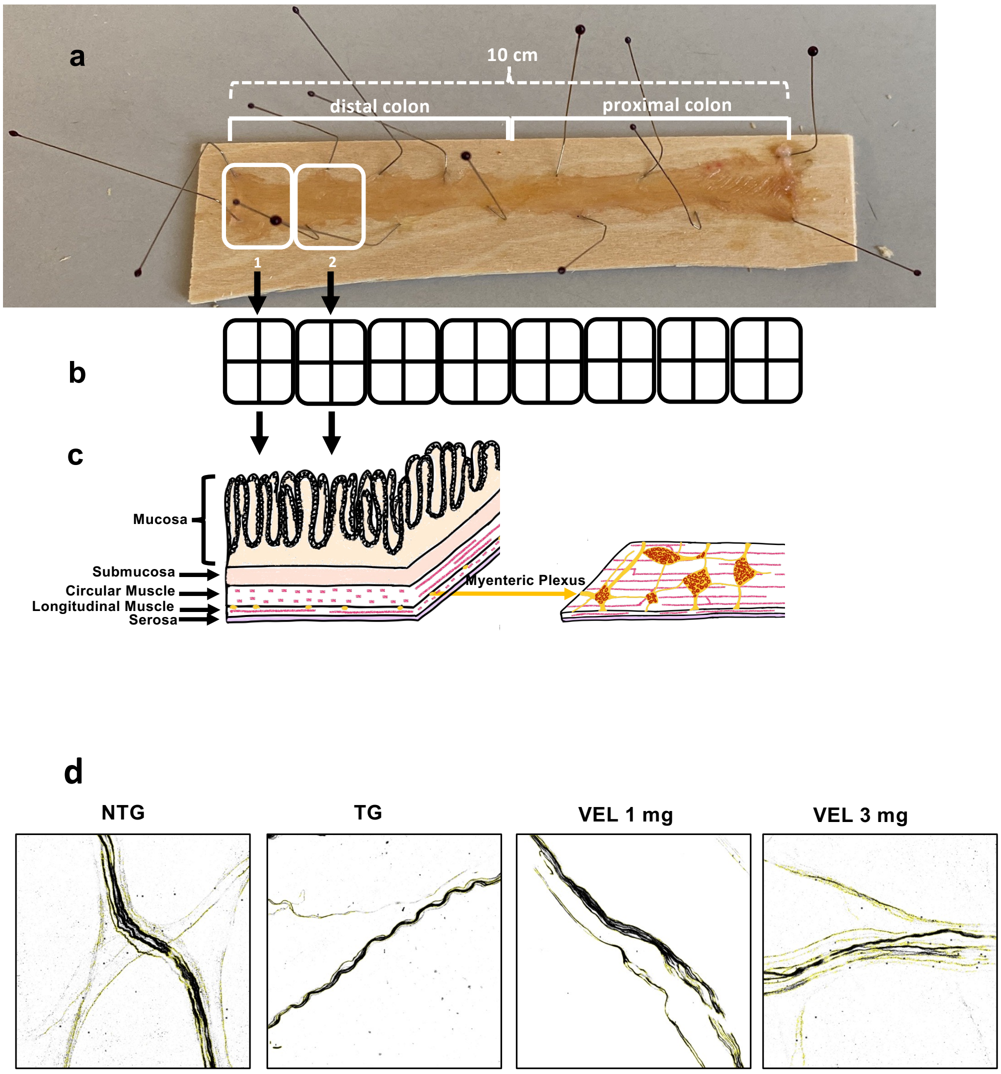

Supplementary Fig 2 Colon dissection scheme for whole mount staining.

a The colon of 6 months old was opened longitudinally and pinned with the mucosa side up on a wafer-thin balsa wood, showing the entire colonic length, which can range from approximately 9 to 10.5 cm. **b** After 24 h fixation with 4% PFA at 4°C, the entire colon was cut into 8 sections of the same size (about 1 cm long), numbered from the 1st to the 8th starting from the distal colon, so that for all colon analyzed, the sectioning pattern always starts from the same point, i.e. section n.1 of the distal colon (the one nearest the anus), and always ends with the number 8 corresponding to the last section of the proximal colon (cecum). Each section was additionally divided into 4 squared subsections and stored in 0.1% NaN_3 in PBS at 4°C for further immunostaining. Immunohistochemistry analyses were performed in the 4 subsections of sections 1, 2 and 3. **c** Organization of the mouse colon. The myenteric

plexus is placed between the longitudinal muscle layer (outer) and the circular muscle (inner). **d** Examples of the approach 2 – "threshold method" used to determine the mean fluorescence intensity for NF-H. Automated selection of region of interest to be quantified is highlighted in yellow. Same approach was also used for VAChT and TH immunostaining.

a

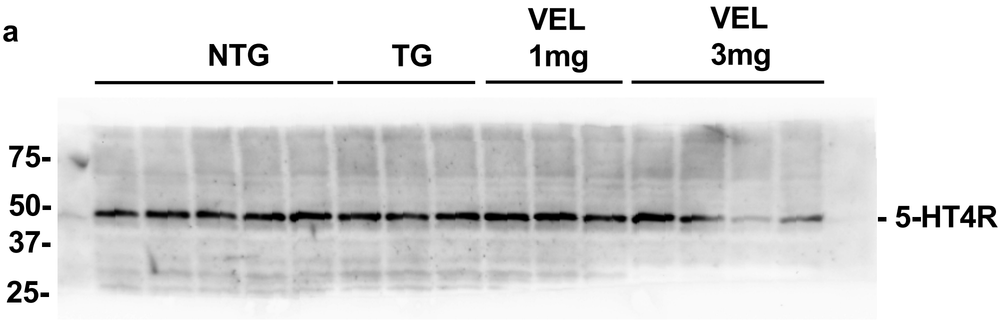

b

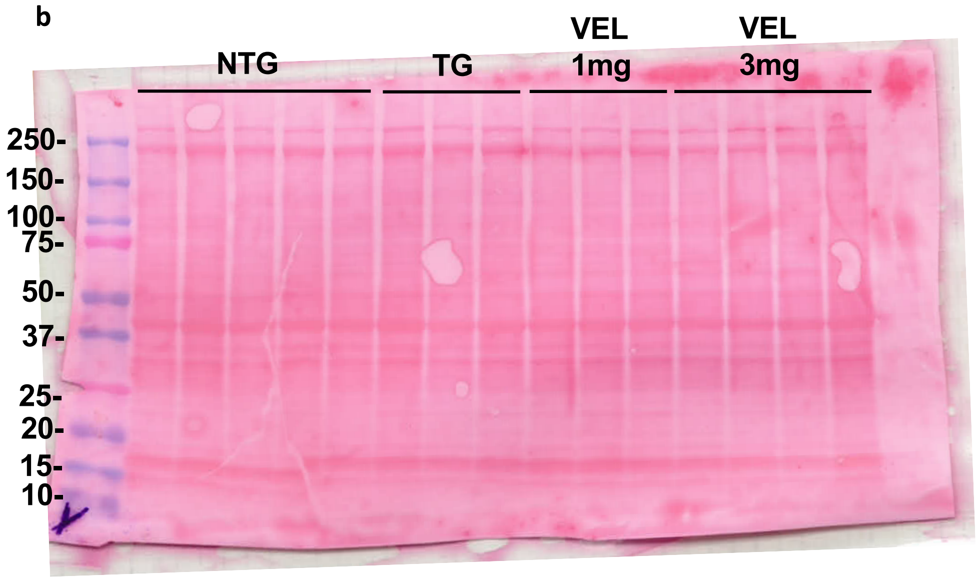

Supplementary Fig 3 Full length Western blots shown in Fig. 2 panel e. Unprocessed, original blot immunoreacted with 5-HT4R antibody (**a**) and ponceau staining (**b**). Samples are total lysates from distal colon of Ntg mice treated with vehicle and age-matches Tgs treated with either vehicle, or 1 or 3 mg Velusetrag.

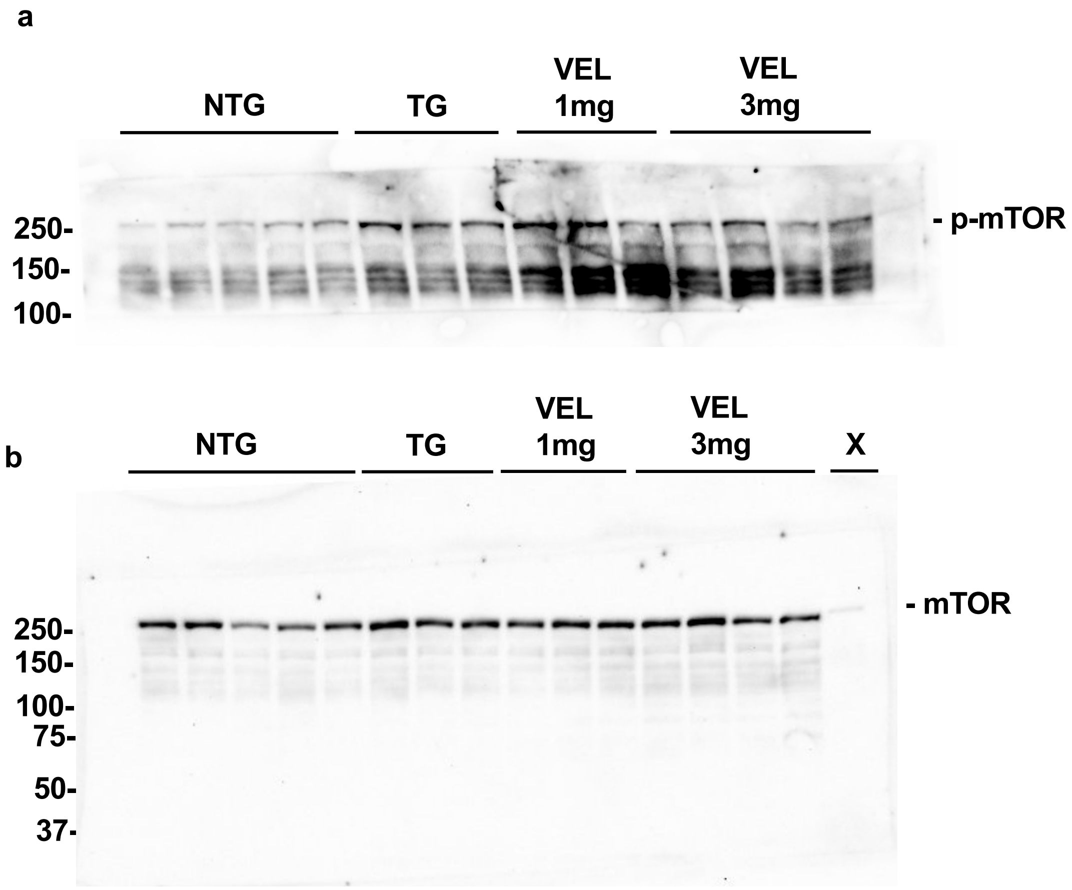

Supplementary Fig 4 Full length Western blots shown in Fig. 4 panel a. Unprocessed, original blots immunoreacted with p-mTOR (**a**) and mTOR antibody (**b**). Samples are total lysates from distal colon of Ntg mice treated with vehicle and age-matches Tgs treated with either vehicle, or 1 or 3 mg Velusetrag. X in blot b= positive control (cell extract).

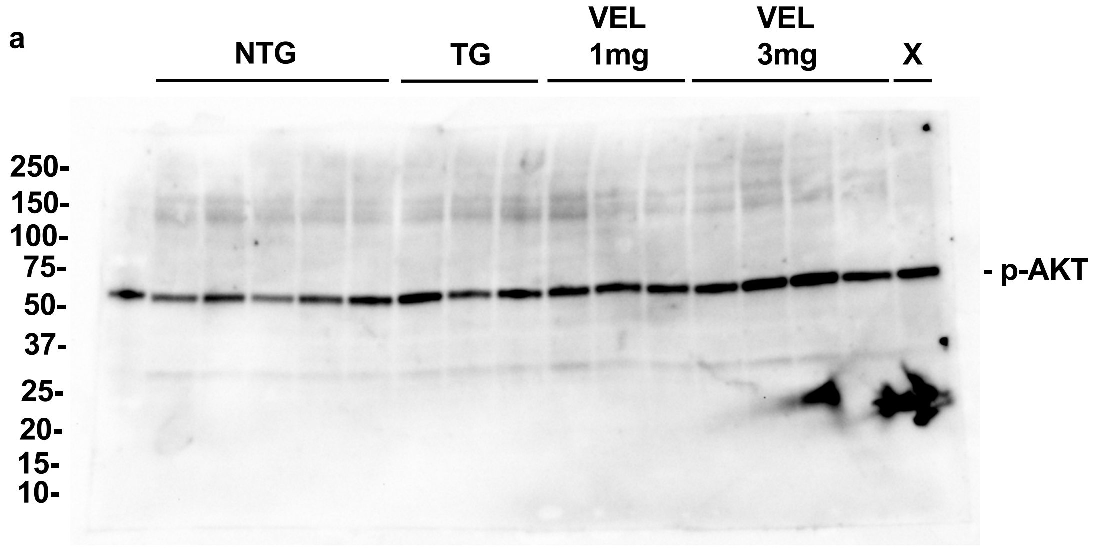

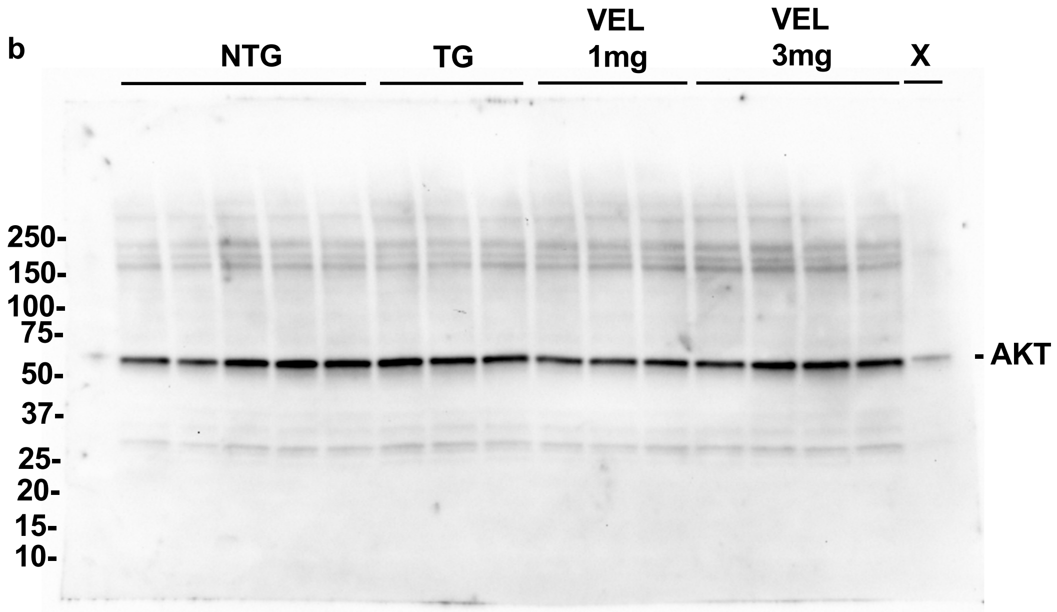

Supplementary Fig 5 Full length Western blots shown in Fig. 4 panel a. Unprocessed, original blots immunoreacted with p-AKT (**a**) and AKT antibody (**b**). Samples are total lysates from distal colon of Ntg mice treated with vehicle and age-matches Tgs treated with either vehicle, or 1 or 3 mg Velusetrag. X in blot a,b= positive control (cell extract).

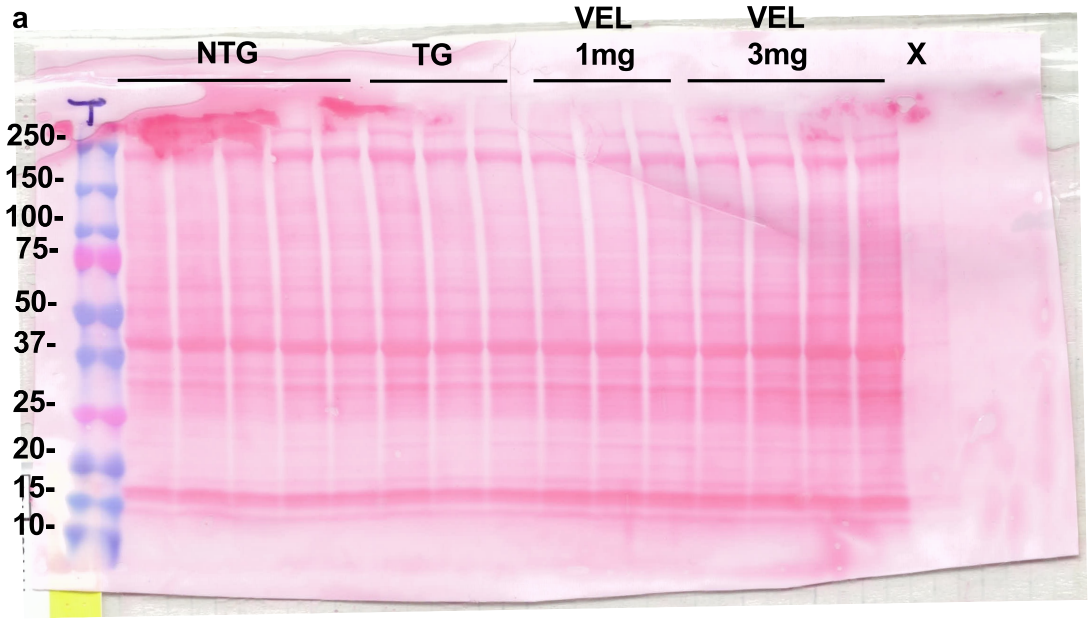

Supplementary Fig 6 Full length Western blots shown in Fig. 4 panel a. Unprocessed, original ponceau staining for blots in figure 4 panel **a**. Samples are total lysates from distal colon of Ntg mice treated with vehicle and age-matches Tgs treated with either vehicle, or 1 or 3 mg Velusetrag. X = positive control (cell extract).

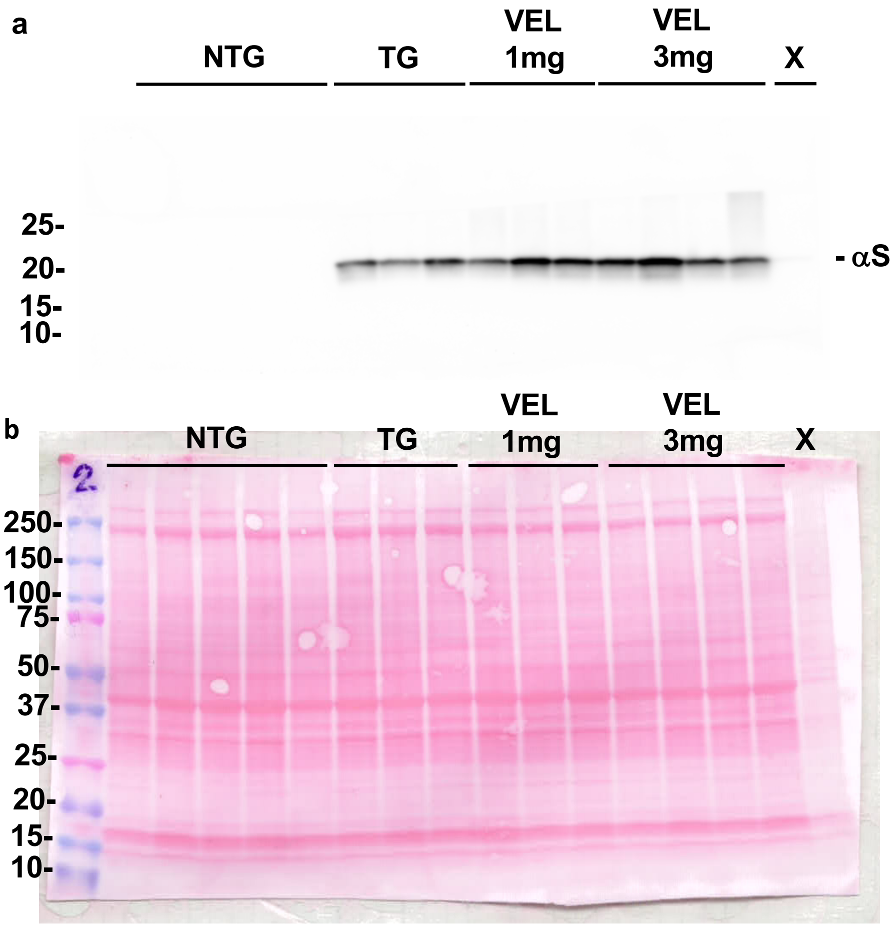

Supplementary Fig 7 Full length Western blots shown in Fig. 6 panel a. Unprocessed, original blot immunoreacted with Syn-1 antibody (**a**) and ponceau staining (**b**). Samples are total lysates from distal colon of Ntg mice treated with vehicle and age-matches Tgs treated with either vehicle, or 1 or 3 mg Velusetrag. X = positive control (cell extract).

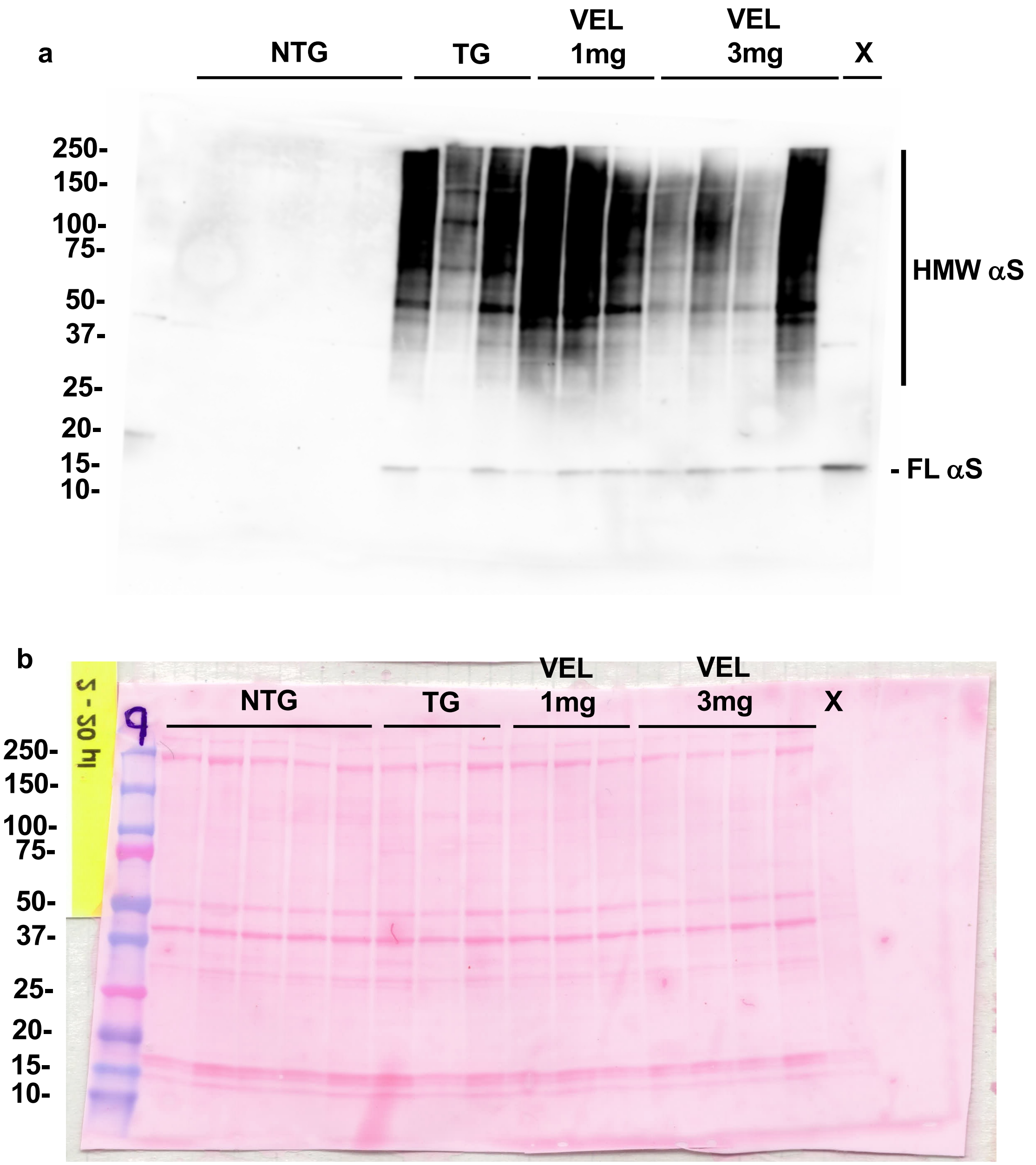

Supplementary Fig 8 Full length Western blot shown in Fig. 6 panel b. Unprocessed, original blot immunoreacted with Syn-1 antibody (**a**) and ponceau staining (**b**) Samples are detergent-insoluble fractions from distal colon of Ntg mice treated with vehicle and age-matches Tgs treated with either vehicle, or 1 or 3 mg Velusetrag. X=positive control (cell extract). FL αS=Full length a-synuclein. HMW αS = high molecular weight αS.
